# Slayton on Gutta Percha

**Published:** 1855-10

**Authors:** N. B. Slayton


### GUTTA PERCHA.

The following communication, from Dr. Slayton, was received a few days since, and accompanying it some three specimens of the mode of putting up work with the material. We have no doubt, from experiments heretofore made in England, as well as in this country, that the Gutta Percha can be made to answer for temporary work. We have known of work worn from one to four years, made of Gutta Percha—in these, however, the coloring matter had all disappeared. The coloring, as done by Dr. Slayton, appears diffused through the entire substance, and of a permanent character.

The specimens sent us by the Doctor are very neat and light—one made entirely of Gutta Percha, with a bar (of silver, we presume,) and base work to which the teeth are attached. The Gutta Percha hides all this and is moulded up around the teeth, looking very natural indeed. One is a metallic plate rimmed inside and out, and the teeth united bv the Gutta Percha, looking like continuous gum work. This style of work has one objection, which is that the material although fitting neatly to the rim does not adhere to it. The other has an outer rim of metal and a base plate of the same, but covered, entirely with the material except the outer rim. The same objection exists here as in the last named, the Gutta Percha does not adhere to the rim. As specimens showing what can be done, they are, however, very neat. For upper plates to be retained by suction, they must be made three or four times as thick as gold; even then that which covers the palate can be easily bent, yet it springs immediately back to its place. This property of the Gutta Percha we are not sure but that it is one of great advantage, adapting the denture to the alveolar arch, during mastication, and preventing loosening of the teeth on the side not being used. For under sets we feel assured that it will be of great use—particularly for temporary work on tender and irritable gums.

In conclusion we would say, of this what the Mississippi Valley Association said of Dr. Allen's continuous gum work—"It is worthy the attention of the Profession." And that the coloring which Dr. Slayton has succeeded in giving it, adds very much to its beauty and value, and appears to accomplish what has for some time been desired. ED.

MADISON, (Ind.) Sept. 20, 1855.

MR. EDITOR:

*Sir:*—I wish to give you the result of a course of experiments that I have been making for the last year with Gutta Percha.

For the last six years considerable attention has been given by dentists to that material to be used as a base for artificial teeth, more especially for temporary work. It was for this purpose that I first commenced using it. My first experiment was with the common Gutta Percha of commerce; not then knowing but that all the Gutta Percha was alike. This, I found by repeated trial, would not answer my purpose, there being various objections to its use; such as color, taste and smell; but the most serious of all, is that it would soften from 115 to 120 deg, making it almost impossible for those who are in the habit of using hot foods and drinks to use it, as it would slide off like wax. After repeated trials and as many partial failures, I commenced a correspondence with the Pressident of the Gutta Percha Company, New York, and from him I learned that there were several varieties of Gutta Percha, and that they differed materially from each other; and that the Gutta Percha now in market was NOT pure, and that I could not make it answer my purpose.

He stated that there was a kind, (if I could get it,) he was satisfied would answer my purpose, but thought it doubtful about my obtaining it.

I immediately visited New York for that purpose, and, after three days' search I found about one and a half pounds. This I found to be a different article from anything that I had ever seen, and in fact it had no resemblance to Gutta Percha that we see in market. With this article I commenced a course of chemical experiments, and the result is, that I have obtained an article that is purely white and semi-transparent. It is of a hard elastic nature—very tenaceous. It has no taste or smell, and it requires a heat as high as 180 degrees to mould it to your plaster model.

My next effort was to take this white Gutta Percha and give it a permanent color. In this I have been equally as successful as the other. I can give it any shade or color I choose, from the deepest to the most delicate shade, and this color will not fade in the least by any process that I have yet been able to apply to it. You can boil it in the strongest alkalies or acids, with no perceptible effect, or you can chew a piece for twenty-four hours and make no impression on the colors. Tobacco will stain it, and cannot easily be removed, but I have found nothing else that appears to have any impression. There may, however, be many things that will stain it, but that is the only thing I have found that would do it.

From this article I am able to make beautiful sets of teeth. I think it looks prettier in the mouth than any gum teeth or block work that I have ever seen. You can make it as firm and as strong as the best plate work. It requires little over an ounce of the Gutta Percha to make a full set.

The teeth that I use with this article is Allen's continuous gum teeth. These are not made to suit in every respect, but are better than common flat teeth. Teeth should be made expressly for this work.

I mould my Gutta Percha on the plaster model and if that is correct, I am certain of a *perfect* fit. With no warping or springing of plate, no irritation of the gums, no falling off the plate in the mouth, and in fact you give better satisfaction to your patient than you possibly can do with a plate. I can make a full set in from six to eight hours, and when finished will cost me about six dollars, it depending on the price that you can obtain the Gutta Percha at. The only question that now arises in my mind as to its being used for permanent work is the length of time that it will wear by actual use. As for temporary work no one can have any doubt as to its preference over anything else that can be used, that has ever seen it and the manner of working it.

But whether it will wear long enough for permanent work I can not say, though I am satisfied that every dentist who will use it at all will use it for lower sets, more or less. There is no change of color by wearing, and, thus far, no wear that is perceptible.

I am using it for permanent work. I have some ten sets out, which I am watching carefully. My patients are delighted with them, and say they will not wear anything else. It is soft and easy to the mouth, fits very tight, and looks better than gum teeth, and much cheaper. This kind of Gutta Percha is very difficult to obtain at present; it has only been used in this country for chemical purposes, being too expensive for common use, and no better for the use that is made of it. My manner of preparing it I have placed in the hands of Messrs. Jones, White & Co., of New York and Philadelphia. They will prepare it and furnish it to dentists cheaper than they could obtain the article and make it themselves. They will make an effort to obtain a supply as soon as possible, for those who wish to use the Gutta Percha.

I do not offer this article to the profession for *permanent work*, but for *temporary* there is nothing better. I should advise any one, at first, to use it as *temporary* and give it a *fair* test; it will then be time enough to use it for permanent work. I am not yet done *experimenting* with Gutta Percha. I am satisfied in my own mind, that it yet will take the place of plate in most every case, and be made equally as *durable*.

Yours, respectfully, N. B. SLAYTON.

